# Src-like adaptor protein 2 (SLAP2) binds to and inhibits FLT3 signaling

**DOI:** 10.18632/oncotarget.10760

**Published:** 2016-07-21

**Authors:** Sausan A. Moharram, Rohit A. Chougule, Xianwei Su, Tianfeng Li, Jianmin Sun, Hui Zhao, Lars Rönnstrand, Julhash U. Kazi

**Affiliations:** ^1^ Division of Translational Cancer Research, Department of Laboratory Medicine, Lund University, Lund, Sweden; ^2^ Lund Stem Cell Center, Department of Laboratory Medicine, Lund University, Lund, Sweden; ^3^ Translational Cancer Research, Lund University, Skåne University Hospital, Department of Oncology, Lund, Sweden; ^4^ Department of Surgery, Faculty of Medicine, The Chinese University of Hong Kong, Hong Kong; ^5^ School of Biomedical Sciences, Faculty of Medicine, The Chinese University of Hong Kong, Hong Kong; ^6^ Department of Pathogen Biology and Immunology, School of Basic Medical Sciences, Ningxia Medical University, Yinchuan, P. R. China

**Keywords:** SLA2, Ba/F3, 32D, AML, AKT

## Abstract

Fms-like tyrosine kinase (FLT3) is a frequently mutated oncogene in acute myeloid leukemia (AML). FLT3 inhibitors display promising results in a clinical setting, but patients relapse after short-term treatment due to the development of resistant disease. Therefore, a better understanding of FLT3 downstream signal transduction pathways will help to identify an alternative target for the treatment of AML patients carrying oncogenic FLT3. Activation of FLT3 results in phosphorylation of FLT3 on several tyrosine residues that recruit SH2 domain-containing signaling proteins. We screened a panel of SH2 domain-containing proteins and identified SLAP2 as a potent interacting partner of FLT3. We demonstrated that interaction occurs when FLT3 is activated, and also, an intact SH2 domain of SLAP2 is required for binding. SLAP2 binding sites in FLT3 mainly overlap with those of SRC. SLAP2 over expression in murine proB cells or myeloid cells inhibited oncogenic FLT3-ITD-mediated cell proliferation and colony formation *in vitro*, and tumor formation *in vivo*. Microarray analysis suggests that higher SLAP2 expression correlates with a gene signature similar to that of loss of oncogene function. Furthermore, FLT3-ITD positive AML patients with higher SLAP2 expression displayed better prognosis compared to those with lower expression of SLAP2. Expression of SLAP2 blocked FLT3 downstream signaling cascades including AKT, ERK, p38 and STAT5. Finally, SLAP2 accelerated FLT3 degradation through enhanced ubiquitination. Collectively, our data suggest that SLAP2 acts as a negative regulator of FLT3 signaling and therefore, modulation of SLAP2 expression levels may provide an alternative therapeutic approach for FLT3-ITD positive AML.

## INTRODUCTION

Acute myeloid leukemia (AML) is a hematopoietic disorder originating from the myeloid lineage [1]. Multiple genetic alterations including loss-of-function mutations in transcription factors and gain-of-function mutations in receptor tyrosine kinases are well-known genetic changes in AML. Among many mutated genes, FLT3 is the most frequently identified oncogene in AML [2]. FLT3 belongs to the type III receptor tyrosine kinase family with the other members including PDGFRA, PDGFRB, KIT, and CSF1R. Key features of this family of proteins include an extracellular ligand-binding domain, a transmembrane domain, a juxtamembrane domain and a kinase domain separated by a short kinase insert. Ligand binding to the receptor induces dimerization of the receptor and autophosphorylation on several tyrosine residues creating docking sites for predominantly SH2 domain-containing signaling proteins. Binding of signaling proteins to the activated receptor results, depending on the binding partner, in activation or inhibition of downstream signaling cascades. For example, association of GRB10, SYK and SRC family kinases to FLT3 enhances downstream signaling, while SOCS2, SOCS6, CSK and LNK negatively regulate FLT3 signaling [3–9]. Therefore, identification of novel interacting proteins will enhance our understanding of FLT3 downstream signaling and will provide an alternative approach to development of therapy.

Although wild-type FLT3 needs its ligand (FL) for activation, oncogenic FLT3 mutants are constitutively active. The most common mutation identified in FLT3 is the internal tandem duplication (ITD) mutation in the juxtamembrane domain. ITD mutation is found in around 25% of AML patients [10]. Even though the ITD mutation commonly occurs in the juxtamembrane domain, recent studies suggest that the duplication can occur in the tyrosine kinase domain as well. The other less frequently occurring mutations include point mutations in kinase domain such as D835X. FLT3-ITD mutations constitutively activate survival and proliferative pathways and confer poor prognosis in AML [11]. Wild-type FLT3 is upon FL stimulation capable of activation of multiple signaling pathways, including the PI3K/AKT, RAS/ERK, and p38 pathways [12–14]. In addition to activation of those pathways, FLT3-ITD activates STAT5 signaling [15].

SRC-like adaptor protein (SLAP) and its homolog SLAP2 are adaptor proteins structurally similar to the SRC family kinases [16]. Like SRC, both SLAP and SLAP2 contain an SRC homology 2 (SH2) domain and an SRC homology 3 (SH3) domain but lack a kinase domain. The presence of SH2 and SH3 domains facilitates association of multiple proteins. For example, SLAP associates with the type III receptor tyrosine kinases FLT3 [17], KIT [18], PDGFRB [19] and CSF1R [20] upon ligand stimulation. The interaction occurs most likely through binding of phosphorylated tyrosine residues in the receptor to the SH2 domain of SLAP. SLAP2 has been shown to associate with CSF1R through its SH2 domain which results in downregulation of the receptor [20]. However, whether SLAP2 plays a role in other type III receptor tyrosine kinases remains unknown. SLAP2 expression has been described in a variety of tissues including thymocytes, leukocytes, lung, spleen, platelets, monocytes, T cells and B cells [16]. Therefore, it is likely that SLAP2 plays a role in hematological malignancies such as AML.

In this study, we identified SLAP2 as an FLT3 interacting protein. SLAP2 associated with ligand-stimulated FLT3 through its SH2 domain. SLAP2 binding sites in FLT3 overlap with the SRC binding sites. Expression of SLAP2 controls oncogenic FLT3-ITD-induced cell proliferation, colony and tumor formation through regulation of AKT, ERK, p38 and STAT5 signaling.

## RESULTS

### SLAP2 associates with FLT3 in response to FL stimulation

**Figure 1:**
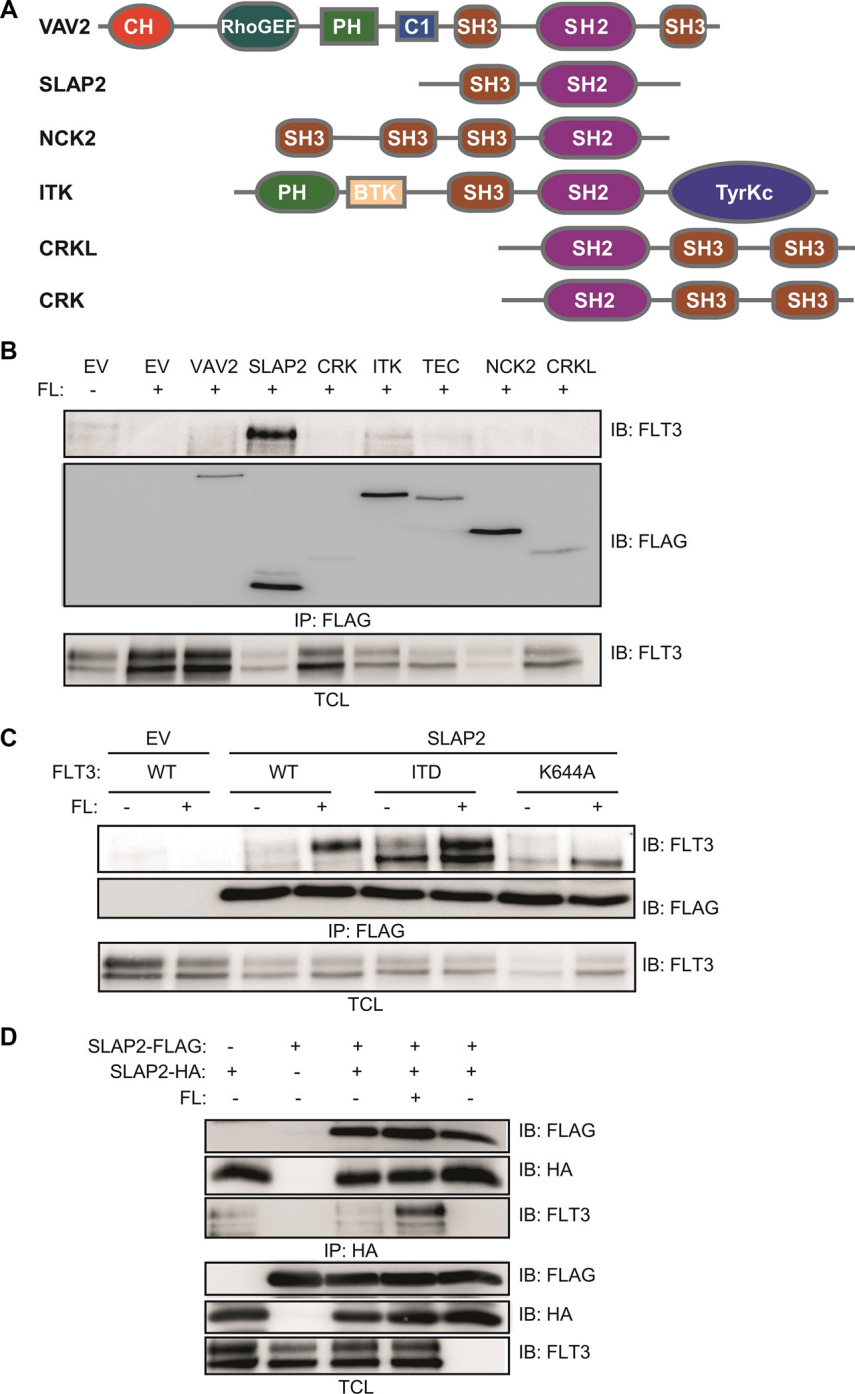
SLAP2 associated with FLT3 in response to FL (**A**) Structure of several SH2 domain-containing proteins. (**B**–**C**) COS-1 cells were transfected with plasmids expressing FLT3-WT and empty vector or VAV2 or SLAP2 or CRK or ITK or TEC or NCK or CRKL (B). COS-1 cells were transfected with plasmids expressing FLT3-WT or FLT3-ITD or FLT3-K644A and empty vector or SLAP2 (C). One day after transfection cells were stimulated for 5 minutes with 100 ng/ml FL followed by lysis. Lysates were subjected to anti-FLAG immunoprecipitations. (**D**) COS-1 cells were transfected with plasmids expressing SLAP2-FLAG and SLAP2-HA. One day after transfection cells were stimulated for 5 minutes with 100 ng/ml FL followed by lysis. Lysates were subjected to anti-HA immunoprecipitations.

To identify novel FLT3 interacting proteins, we tested seven different SH2 domain-containing proteins including VAV2, SLAP2, CRK, ITK, TEC, NCK2 and CRKL (Figure 1A). SH2 domain is a phosphotyrosine binding domain and after activation, several tyrosine residues of FLT3 become phosphorylated. Thus, it is likely that SH2 domain-containing proteins will associate with FLT3 upon activation. COS-1 cells were transfected with plasmids for FLT3-WT or FLAG-tagged VAV2, SLAP2, CRK, ITK, TEC, NCK2, CRKL and empty vector. One day after transfection cells were stimulated with 100 ng/ml FL before lysis. Cell lysates were immunoprecipitated using 1 μg/ml anti-FLAG antibody and then analyzed by Western blotting. We observed a strong association of SLAP2 with ligand-stimulated FLT3 (Figure 1B). VAV2, ITK, TEC, and NCK2 displayed weaker interaction compared to that of SLAP2. Association of SLAP2 with wild-type FLT3 was dependent on FL stimulation (Figure 1C). Oncogenic FLT3-ITD showed constitutive association with SLAP2 while a kinase-dead mutant of FLT3, FLT3-K644A, did not bind to SLAP2. These results suggest that the interaction between FLT3 and SLAP is dependent on FLT3 activation, in other words, tyrosine phosphorylation of FLT3. Additionally, using HA- and FLAG-tagged SLAP2 plasmids we showed that SLAP2-HA and SLAP2-FLAG form a complex (Figure 1D). However, complex formation was not dependent on the expression of FLT3 or of ligand stimulation.

### SLAP2 SH2 domain is involved in association with FLT3 mainly through pY589 and pY591

**Figure 2:**
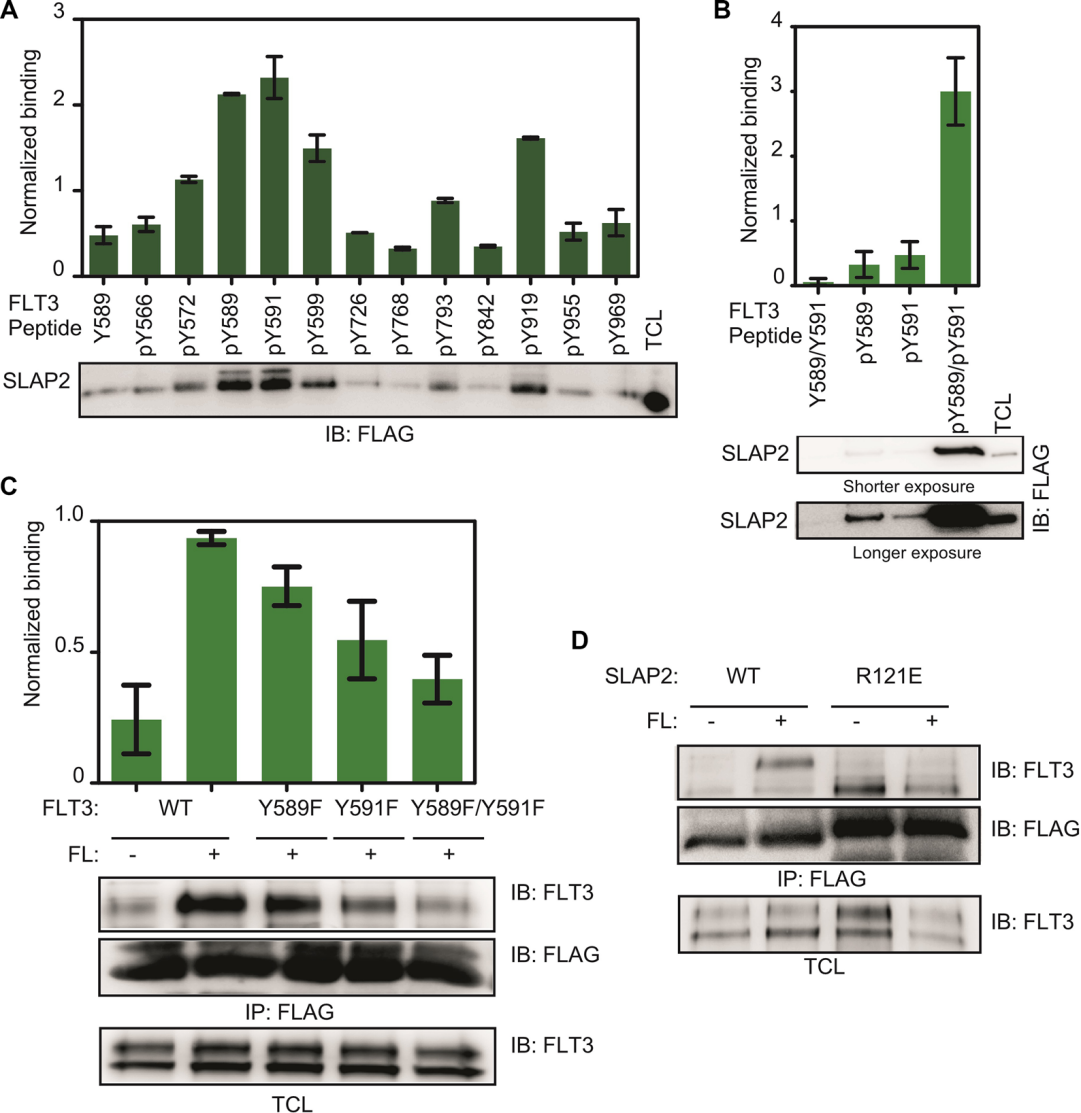
SLAP2 SH2 domain associated with FLT3 phospho-tyrosine residues (**A**–**B**) COS-1 cells were transfected with a plasmid expressing SLAP2-FLAG. Cells were lysed one day after transfection and lysates were mixed with immobilized FLT3 phosphopeptides for 30 minutes. Beads were then washed with lysis buffer for four times and subjected to Western blotting analysis, (**C**–**D**) COS-1 cells were transfected with plasmids expressing SLAP2-FLAG and FLT3-WT or FLT3-WT-Y589F or FLT3-WT-Y591F or FLT3-WT-Y589F/Y591F (C). COS-1 cells were transfected with plasmids expressing FLT3-WT or SLAP2-WT-FLAG or SLAP2-R121E-FLAG (D) One day after transfection cells were stimulated for 5 minutes with 100 ng/ml FL followed by lysis. Lysates were subjected to anti-FLAG immunoprecipitations.

Upon ligand stimulation, several tyrosine residues in the intracellular domain of FLT3 become tyrosine phosphorylated [21] which in turn recruit SH2 domain-containing signaling proteins. To identify the probable SLAP2 binding sites in FLT3, we used phosphopeptides corresponding to twelve FLT3 tyrosine phosphorylation sites. Multiple FLT3 phosphopeptides were able to pull-down SLAP2 from cell lysates (Figure 2A). Peptides corresponding to pY589 and pY591 displayed comparatively stronger association while other binding sites included pY599 and pY919. Furthermore, a doubly phosphorylated peptide, pY589/pY591, showed dramatically increased affinity compared to either pY589 and pY591 alone (Figure 2B) and, similarly, mutations in these tyrosine residues significantly decreased association (Figure 2C). Thus, it is likely that SLAP2 has multiple binding sites in FLT3 with the two major binding sites being pY589 and pY591. To explore whether the SLAP2 SH2 domain is involved in association with the phospho-tyrosine residues, we generated an SH2 domain mutant of SLAP2. The positively charged critical arginine residue (Arg 121) in the SH2 domain was replaced with a negatively charged glutamic acid residue. This mutation completely abolished FLT3 and SLAP2 interaction (Figure 2D) suggesting that a functional SLAP2 SH2 domain is required for the interaction with FLT3.

### SLAP2 expression reduced cell proliferation but did not affect cell survival

**Figure 3:**
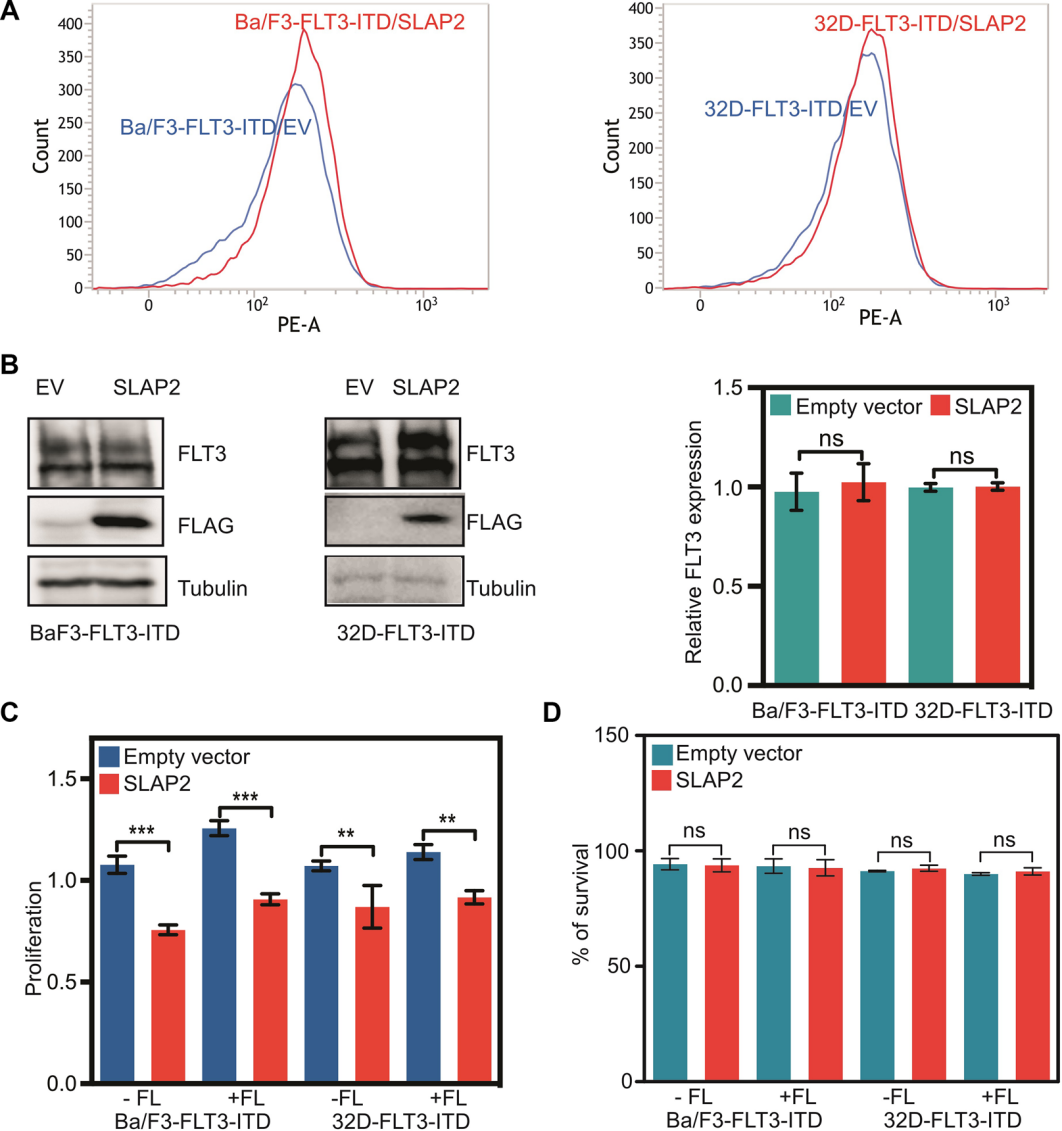
SLAP2 expression inhibits FLT3-ITD mediated cell proliferation (**A**) Ba/F3 and 32D cells expressing FLT3 and SLAP2, or empty vector were labeled with phycoerythrin-conjugated anti-FLT3 and then analyzed by flow cytometry. (**B**) An equal amount of cells were lysed, and lysates were subjected to Western blotting analysis. Blots from multiple experiments were quantified for statistical analysis. ns, not significant. (**C**) Cells were washed to remove IL3 and seeded in a 96-well plate, without FL or IL3, with FL, and with IL3. Two days after seeding cells were subjected to PrestoBlue assay. IL3 containing cells were used for normalization. (**D**) Cells negative for Annexin V and 7-aminoactinomycin D (7-AAD) were counted as survival cells. ns, not significant; **p < 0.01; ***p < 0.001.

Expression of oncogenic FLT3-ITD in murine pro-B, Ba/F3 or myeloid, 32D cells can support cytokine-independent cell survival and transformation. To explore whether the interaction of SLAP2 plays a role in FLT3-ITD induced cell survival and transformation, we overexpressed FLT3-ITD and SLAP2 or empty vector in Ba/F3 and 32D cells. Cell surface expression of FLT3 was detected using flow cytometry and found to be equal in both empty vector and SLAP2 transfected cell lines (Figure 3A). Total FLT3 expression and SLAP2 expression were detected by Western blotting and quantification of multiple blots showed no difference of total FLT3-ITD expression in empty vector and SLAP2 transfected cells (Figure 3B). Expression of SLAP2 significantly reduced FLT3-ITD-dependent cell proliferation in both Ba/F3 and 32D cell lines (Figure 3C). However, overexpression of SLAP2 did not increase cell death upon cytokine depletion (Figure 3D).

### SLAP2 expression reduced *in vitro* colony formation and delayed *in vivo* tumor formation

**Figure 4:**
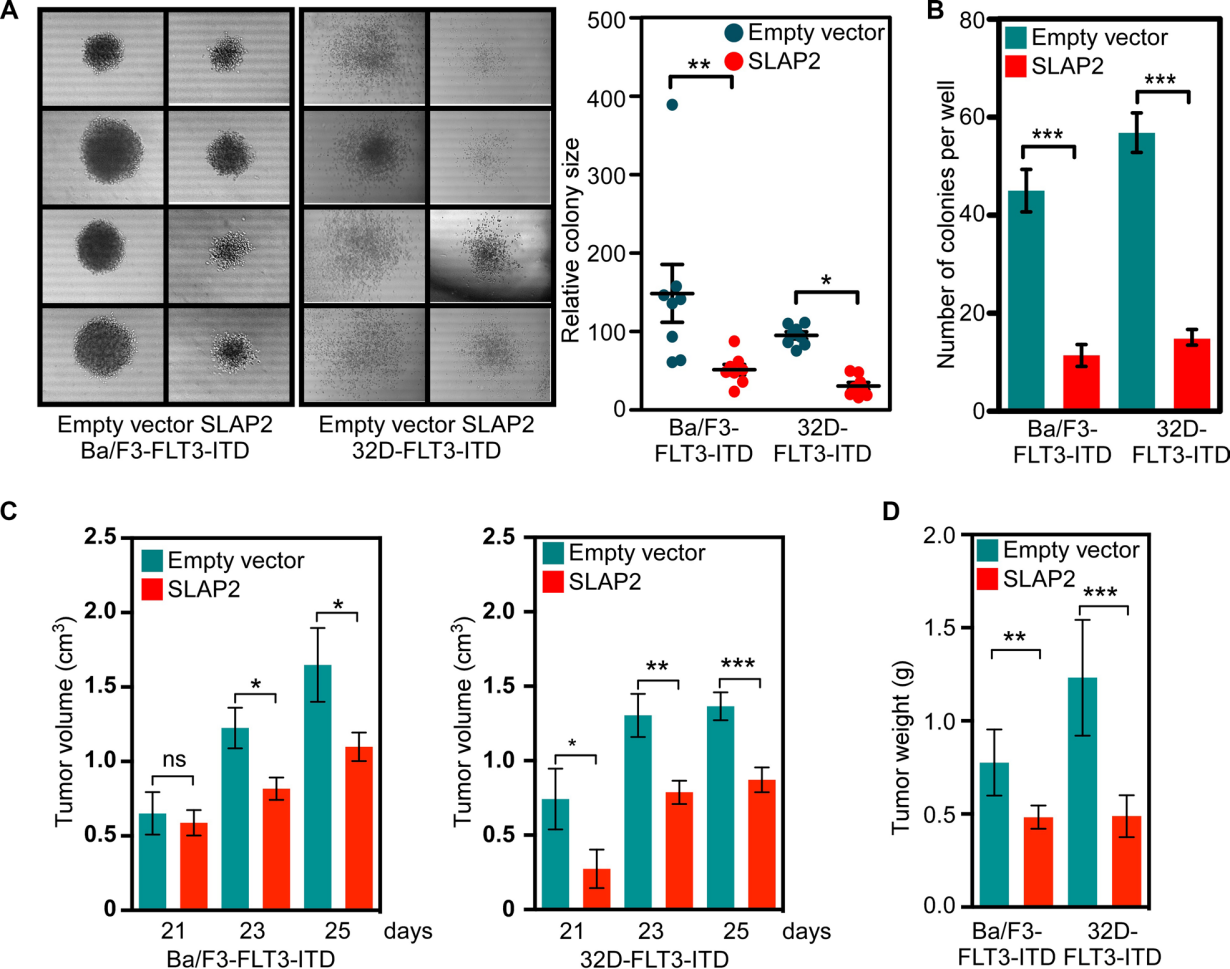
SLAP2 expression reduces FLT3-induced colony formation and tumor formation (**A**–**B**) Cells were washed to remove IL3 and then mixed with methylcellulose medium before seeding in a 24-well plate. Cells were incubated for 7 days. Area of colonies was measured using ImageJ (**C**–**D**) Cells were washed and xenografted into mice. Tumor growth was monitored for 25 days. ns, not significant; *p < 0.05; **p < 0.01; ***p < 0.001.

To understand the role of SLAP2 in FLT3-ITD mediated cellular transformation we used colony formation assays in semi-solid medium and tumor formation capacity in xenografted mice. We observed that expression of SLAP2 significantly decreased colony size (Figure 4A). The number of colonies per well of a 24-well plate was also reduced significantly (Figure 4B) suggesting that cells expressing SLAP2 suppress the FLT3-ITD induced oncogenic potential. To further address this issue we developed a mouse xenograft model using immunocompromised mice. Expression of SLAP2 significantly decreased tumor volume (Figure 4C) as well as tumor weight (Figure 4D) in xenografted mice.

### SLAP2 expression controls oncogenic signaling

**Figure 5:**
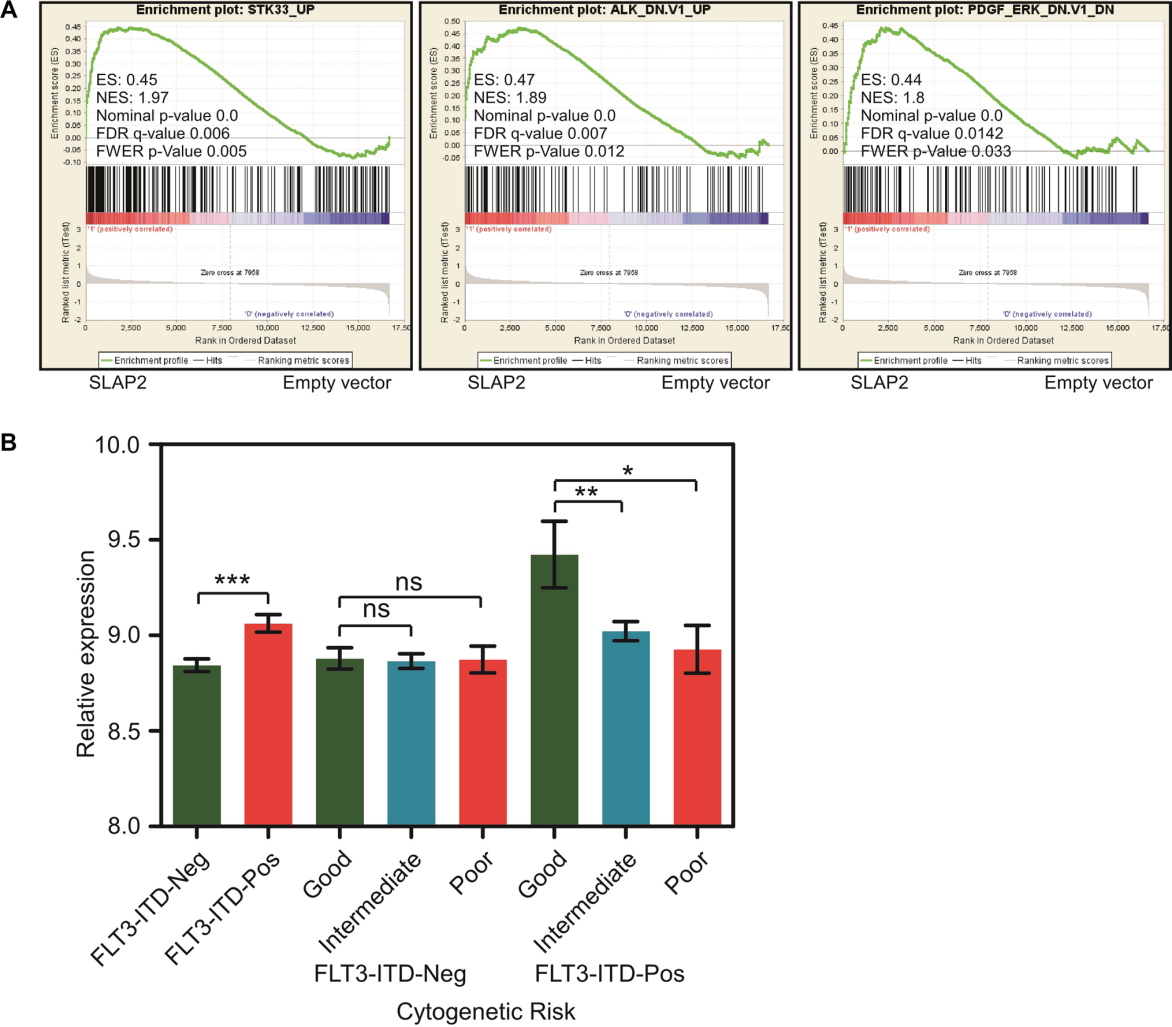
SLAP2 expression led to better survival in FLT3-ITD positive AML (**A**) GESA analysis using mRNA expression of Ba/F3 and 32D cells expressing SLAP2 or empty vector. (**B**) Relative SLAP2 expression in FLT3-ITD positive or negative AML patients. ns, not significant; *p < 0.05; **p < 0.01; ***p < 0.001.

Then we checked whether SLAP2 has a role in FLT3-ITD-induced gene expression. We used microarray to compare mRNA expression between cells expressing SLAP2 and empty vector. We found that SLAP2 expressing cells have a gene signature that correlates with that of loss of STK33, ALK or PDGFR function (Figure 5A) suggesting that SLAP2 plays a role in controlling oncogenic signals from FLT3-ITD. In addition, using AML patient data, we showed that AML patients carrying FLT3-ITD have a significantly enhanced SLAP2 expression and FLT3-ITD positive AML patients with comparatively lower SLAP2 expression have intermediate or poor prognosis (Figure 5B).

### SLAP2 expression partially blocked FLT3 downstream signaling

**Figure 6:**
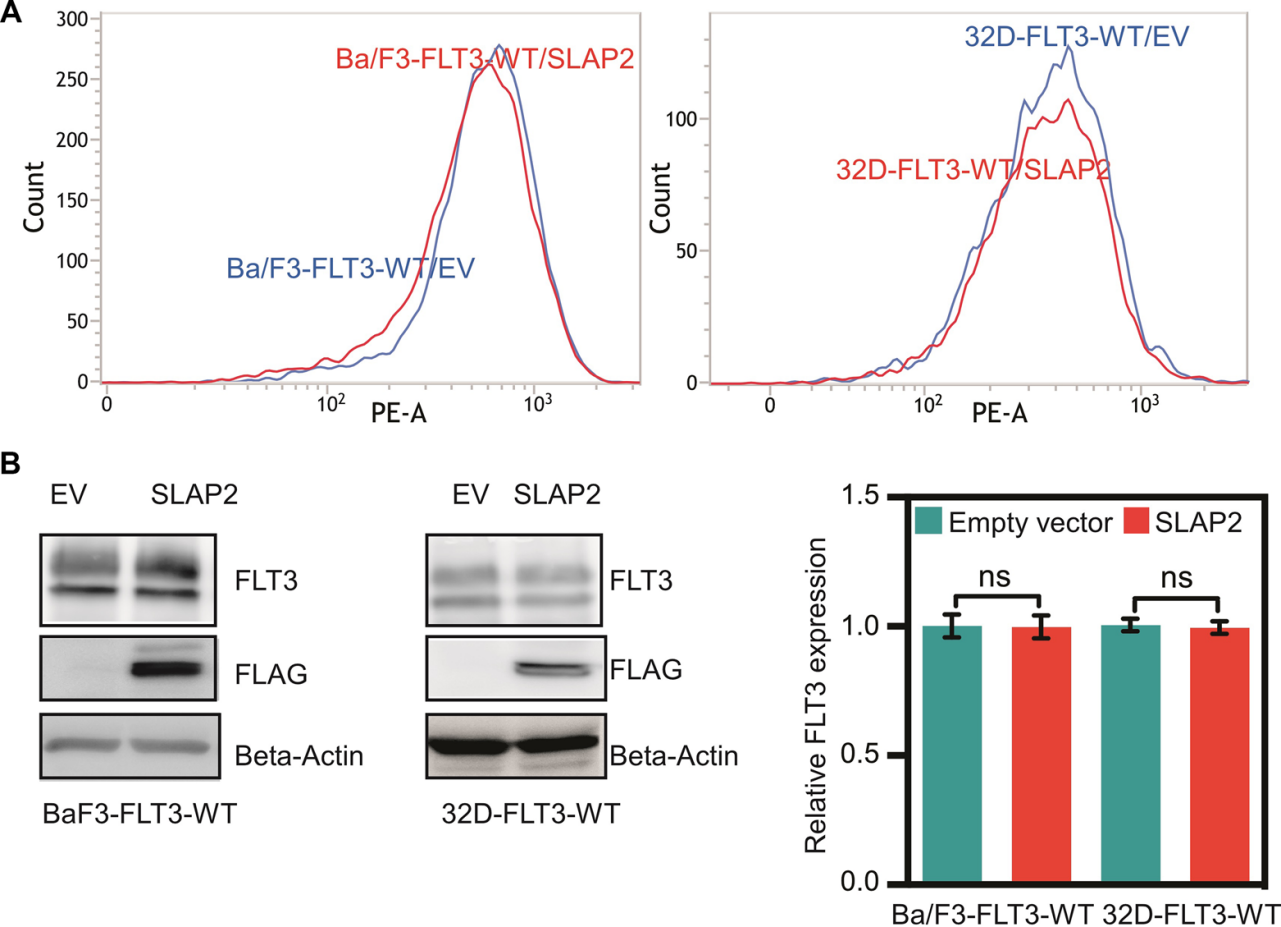
Ba/F3 and 32D cell lines expressing wild-type FLT3 and SLAP2 (**A**) Ba/F3 and 32D cells expressing FLT3 and SLAP2 or empty vector were labeled with phycoerythrin-conjugated anti-FLT3 and then analyzed by flow cytometry. (**B**) An equal amount of cells were lysed, and lysates were subjected to Western blotting analysis. Blots from multiple experiments were quantified for statistical analysis. ns, not significant.

**Figure 7:**
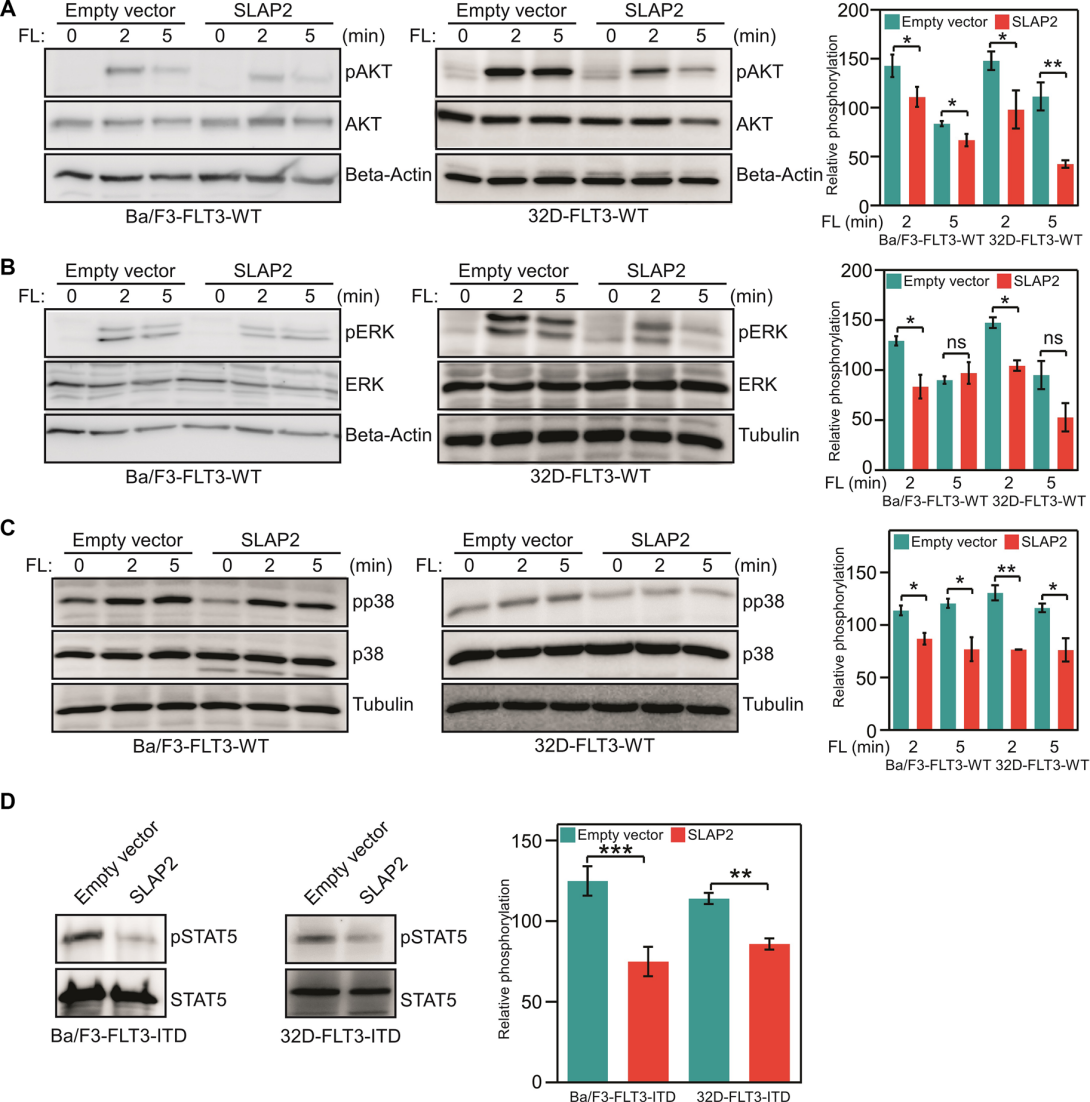
SLAP2 expression suppresses FLT3 downstream signaling (**A**–**C**) Ba/F3 and 32D cells expressing wild-type FLT3, and empty vector or SLAP2 were washed to remove IL3 and starved four hours before FL stimulation. Total cell lysates were used for SDS-PAGE and Western blotting analysis using anti-phospho-AKT (A), anti-phospho-ERK1/2 (B), anti-phospho-p38 (C) antibodies. (**D**) Ba/F3 and 32D cells expressing FLT3-ITD and empty vector or SLAP2 were washed to remove IL3 and starved four hours before FL stimulation. Total cell lysates were subjected to anti-STAT5 immunoprecipitation followed by SDS-PAGE and Western blotting analysis using 4G10 antibody. For statistical analysis multiple blots were quantified. ns, not significant; *p < 0.05; **p < 0.01; ***p < 0.001.

To understand the molecular mechanism of how SLAP2 controls FLT3-mediated biological events we generated Ba/F3 and 32D cell lines stably expressing FLT3-WT and empty control vector or SLAP2. Cell surface expression of FLT3-WT was checked by flow cytometry (Figure 6A) and total FLT3 expression was verified by Western blotting (Figure 6B) demonstrating the same FLT3 expression in all cell lines. Since wild-type FLT3 is dependent on FL for activation, the signal from the receptor can be controlled by ligand stimulation. As described in Introduction, wild-type FLT3 activation results in activation of the PI3K/AKT, ERK, and p38 pathways [13, 14]. Thus, the role of SLAP2 in FLT3 downstream signaling can be monitored by measuring AKT, ERK, and p38 phosphorylation. We demonstrated that SLAP2 expression significantly decreased FLT3-induced AKT phosphorylation (Figure 7A). ERK1/2 phosphorylation was reduced at the 2 minutes time point, but the difference was not statistically significant at 5 minutes of FL stimulation (Figure 7B). Similar to the AKT phosphorylation, p38 phosphorylation was significantly decreased in SLAP2 expressing cells (Figure 7C). Moreover, using Ba/F3 and 32D cells expressing FLT3-ITD and SLAP2 or empty vector we observed that STAT5 phosphorylation was significantly decreased in SLAP2 expressing cells (Figure 7D). Thus, we suggest that SLAP2 regulates FLT3 downstream signaling.

### SLAP2 expression accelerates FLT3 degradation by enhancing ubiquitination

**Figure 8:**
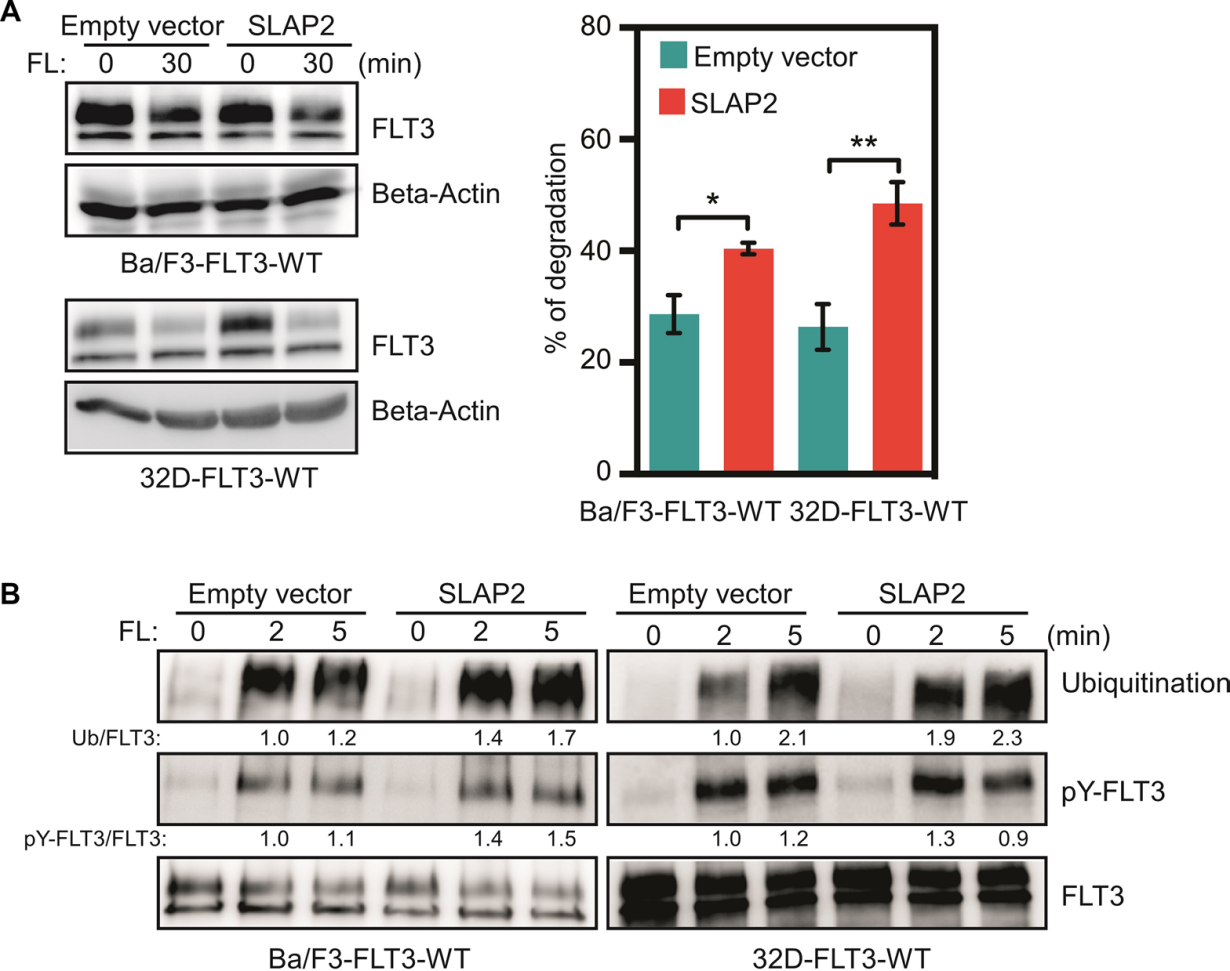
SLAP2 expression accelerated FLT3 degradation through enhanced ubiquitination (**A**) Cells were washed to remove IL3 and incubated with cycloheximide before FL stimulation. Total cell lysates were used for SDS-PAGE and Western blotting analysis. Multiple blots were quantified for statistical analysis. *p < 0.05; **p < 0.01. (**B**) Cells were washed and starved for 4 hours before stimulation with FL for different time points. Cell lysates were subjected to an anti-FLT3 immunoprecipitation followed by SDS-PAGE and Western blotting analysis.

We then asked the question how SLAP2 controls FLT3-induced downstream signaling. In our previous studies, we have shown that SLAP alters FLT3 and KIT ubiquitination and stability [17, 18]. Therefore, we hypothesized that SLAP2 might play a role in regulation of FLT3 stability. We stimulated Ba/F3 cells with FL for 30 minutes in the presence of cycloheximide (an inhibitor of protein synthesis) and calculated the degradation of FLT3. We found that SLAP2 expression significantly accelerated FL-induced receptor degradation (Figure 8A). We then checked whether the accelerated degradation was due to the enhancement of ubiquitination of FLT3 in SLAP2 expressing cells as it has been shown that SLAP2 expression enhances ubiquitination of another type III receptor tyrosine kinase CSF1R [20]. We observed that cells expressing SLAP2 have a 30 to 90% enhancement in FLT3 ubiquitination (Figure 8B) suggesting that SLAP2 expression decreases FLT3 stability through ubiquitination-mediated degradation.

## DISCUSSION

Growth factor receptor signaling is tightly controlled by associating proteins. Associating proteins either potentiate or diminish receptor signaling. In this report, we showed that SLAP2 acts as a negative regulator of FLT3 signaling. We identified SLAP2 as a novel binding partner of both wild-type and an oncogenic mutant of FLT3. We showed that SLAP2 expression controlled FLT3-ITD mediated cell proliferation, colony formation and tumor formation through suppression of FLT3 downstream signaling by destabilizing the receptor.

SLAP2 displayed a higher affinity for FLT3 compared to many other SH2 domain-containing proteins. The FLT3/SLAP2 interaction was FL-dependent, and a kinase-dead FLT3 mutant did not interact with SLAP2 suggesting that FLT3 activation is required for the interaction. Furthermore, an SH2 domain mutant of SLAP2 was unable to bind with FLT3 demonstrating the fact that the SLAP2 SH2 domain associates with FLT3 through phosphotyrosine residues. By means of peptide fishing and co-immunoprecipitation assays using FLT3 double Y-to-F mutants with SLAP2, we identified Y589 and Y591 residues as major determinants for the interaction. A phosphopeptide containing both Y589 and Y591 sites displayed a strong enhancement of binding suggesting that the negative charge on the adjacent site increases binding affinity. This has been shown to be the case in the PDGF receptor association with SRC, where pY579 binds to the SH2 domain of SRC, while pY581 forms an acidic determinant that enhances the affinity of interaction [22]. Other lower affinity interaction sites include Y599 and Y919. Interestingly, three SLAP2 binding sites in FLT3 such as Y589, Y591, and Y599 were reported as SRC binding sites in FLT3 as well [23, 24]. Therefore, it is possible that SLAP2 competes with SRC and loss of SLAP2 expression potentiates FLT3 signaling through SRC. The other site Y919 has not been studied well. It seems that Y919 is a common binding site for negative regulators. We showed Y919 as a binding site for SOCS6 and LNK [8, 25]. The similar residue in murine FLT3 (Y920) has shown to be essential for receptor activation and corresponding residues in KIT (900) and PDGFRB (Y934) were shown to be SRC-dependent phosphorylation sites [26–28].

In normal tissues, FLT3 expression is predominantly restricted to hematopoietic stem and progenitor cells. SLAP2 expression also overlaps with FLT3 expression suggesting a function in normal hematopoiesis [16]. We observed an increase in expression of SLAP2 in AML patients carrying FLT3-ITD mutations and FLT3-ITD positive AML patients having comparatively lower SLAP2 expression suffer from intermediate or poor prognosis suggesting that SLAP2 plays an important role in FLT3-ITD driven AML. Moreover, SLAP2 expression in lymphoid and myeloid cells inhibits FLT3-induced proliferation, colony formation, and tumor formation. Therefore, it is likely that SLAP2 acts as an important negative regulator of FLT3.

The major pathways involved in FLT3-mediated mitogenic signaling include PI3K/AKT, RAS/ERK, p38 and STAT5 pathways [13, 14]. Using phosphospecific antibodies against AKT, ERK1/2, p38 and STAT5 we showed that expression of SLAP2 significantly reduced FLT3-induced activation of all four pathways. Findings demonstrate that SLAP2 acts on receptor directly but not any specific downstream pathways. SRC is involved in activation of the majority of FLT3 downstream pathways and thus, raises a possibility that competition between SRC and SLAP2 for binding to FLT3 determines the activation of downstream signaling. Another possibility could be destabilization of FLT3 by recruitment of CBL. The C-terminal region of SLAP2 has been shown to be a binding site of CBL [20]. SLAP2 recruits CBL to CSF1R resulting in increased ubiquitination followed by degradation of the receptor [20]. Similarly, we observed that SLAP2 increases FLT3 degradation by enhancing ubiquitination. Therefore, we suggest that SLAP2-mediated negative regulation of FLT3 signaling is probably accomplished through two distinctive mechanisms: competition with SRC and destabilization of FLT3.

FLT3 is the most frequently mutated genes in AML and thus an attractive target for the development of drugs for AML treatment. However, drugs targeting FLT3 suffer from a limited response in the clinic. Intracellular delivery of negative regulatory proteins showed promising results in inhibition of leukemic cell growth [29]. Thus, our present study may provide an alternative therapeutic approach for the treatment of oncogenic FLT3-ITD positive AML patients.

## MATERIALS AND METHODS

### Reagents and plasmids

The transfection reagent Lipofectamine 2000 was from Thermo Fisher Scientific. FLT3 ligand (FL) was from ORF Genetics, and cycloheximide was from Sigma-Aldrich. Rabbit anti-FLT3 antibody was described previously [25]. Mouse anti-FLAG (M2) antibody and horseradish peroxidase-coupled anti-FLAG (M2) antibody were from Sigma-Aldrich. Mouse anti-mono-ubiquitin antibody and mouse anti-phosphotyrosine (4G10) antibody were from Covance Research Products and Millipore respectively. Rabbit anti-phospho-AKT (pSer473) was from Epitomics. Rabbit anti-phospho-ERK1/2 (pThr202/pThr204), goat anti-AKT, rabbit anti-STAT5 and rabbit anti-ERK2 were from Santa Cruz Biotechnology. Mouse anti-phospho-p38 and anti-p38 were from BD Transduction Laboratories. The phycoerythrin-labeled anti-FLT3 antibody was from BD Biosciences. Horseradish peroxidase-coupled secondary anti-mouse and anti-rabbit antibodies were from Thermo Scientific, and anti-goat was from Santa Cruz Biotechnology. Expression plasmids pcDNA3-FLT3-WT, pcDNA3-FLT3-ITD, pcDNA3-FLT3-K644A, pcDNA3-FLT3-Y589F, pcDNA3-FLT3-Y591F, pcDNA3-FLT3- Y589/591F, pMSCVpuro-FLT3-WT and pMSCVpuro-FLT3-ITD plasmids were described elsewhere [7]. Murine full-length SLAP2, VAV2, CRK, CRKL, ITK, TEC and NCK2 plasmids in the pCMV-Myc-FLAG vector were obtained from Origene. A plasmid expressing SLAP2 SH2 domain mutant, pCMV-SLAP2-R121E was generated using the QuikChange site-directed mutagenesis kit (Stratagene). HA-tag SLAP2-WT plasmid, pCMV-SLAP2-WT-HA was sub-cloned using the pCMV-HA vector from Origene. Retroviral plasmid pMSCVneo-SLAP2-WT-Myc-FLAG was generated by ligating SLAP-WT-Myc-FLAG fragment to the pMSCVneo vector.

### Cell culture, transient and stable transfection

COS-1, Ba/F3, and 32D cells were purchased from DSMZ. COS-1 cells were maintained in Dulbecco's modified Eagle's medium (DMEM) supplemented with 10% fetal bovine serum (FBS), 100 units/ml penicillin and 100 μg/ml streptomycin. Ba/F3 and 32D cells were cultured in RPMI 1640 medium supplemented with 10% heat-inactivated FBS, 10 ng/ml recombinant murine interleukin-3 (IL3), 100 units/ml penicillin and 100 μg/ml streptomycin. COS-1 cells were used for transient transfection with Lipofectamine 2000 according to the manufacture's protocol. Ba/F3 and 32D cells were used for stable transfections with retroviral vector pMSCV. EcoPack packaging cells were transfected with pMSCVpuro-FLT3-WT and pMSCVpuro-FLT3-ITD constructs using Lipofectamine 2000. Virus-containing supernatants were collected 72 hours after transfection, and both Ba/F3 and 32D cells were infected with virus particles. Cells were then selected using 1.2 μg/ml puromycin. FLT3 expression was confirmed by flow cytometry and Western blotting. EcoPack cells were further transfected with pMSCVneo empty vector or pMSCVneo-SLAP2-WT plasmids, and virus particles were collected for infection of Ba/F3-FLT3-WT, Ba/F3-FLT3-ITD, 32D-FLT3-WT and 32D-FLT3-ITD cells. Cells were then further selected against 0.8 mg/ml G-418 for 2 weeks. SLAP2 expression was verified using Western blotting. All stably transfected cells were maintained in RPMI 1640 medium supplemented with 10% heat-inactivated FBS, 10 ng/ml recombinant murine interleukin-3 (IL3), 100 units/ml penicillin and 100 μg/ml streptomycin as recommended previously [30].

### Immunoprecipitation and Western blotting

Ba/F3 and 32D were washed three times to remove IL3 and serum and starved of serum and cytokines for 4 hours in RPMI-1640. Cells were then stimulated with 100 ng/ml of FL at 37°C followed by washing with ice-cold PBS. Cells were then lysed in Triton X-100 lysis buffer. One μg primary antibody was used for 1 ml cell lysates for immunoprecipitation. The Western blotting procedure was described elsewhere [31]. For immunodetection, Luminata Forte Western HRP Substrate (Millipore) and LAS-3000 CCD camera (Fujifilm) were used. Signal intensity was quantified using Fiji (An ImageJ distribution) and Multi-Gauge (Fujifilm) softwares.

### Affinity fishing of SLAP2 using immobilized FLT3 phosphopeptides

Phosphopeptides, described previously [25], corresponding to known and predicted tyrosine phosphorylation sites in FLT3 intracellular domain were immobilized on UltraLink beads (Thermo Scientific) following the manufacturer's protocol. Twenty microliters of a 1:1 slurry of immobilized peptides was incubated at 4°C for 20 minutes with SLAP2-transfected COS-1 cell lysates. Beads were then washed four times with 500 mM NaCl containing lysis buffer and then processed for Western blotting.

### Cell proliferation, apoptosis, and colony formation assays

Ba/F3 and 32D cells expressing FLT3-ITD were used for cell proliferation, apoptosis and colony formation assays. Cells were washed three times with RPMI 1640 to remove cytokine. Cells were then resuspended in RPMI 1640 supplemented with 10% FBS, 100 units/ml penicillin and 100 μg/ml streptomycin. For cell proliferation assay, cells were seeded in a 96-well plate at a concentration of 10,000 cells per 90 μl. Cells were seeded using three different conditions, with IL3, with FL, and without IL3 or FL. After 46 hours incubation, 10 μl Presto Blue (Molecular Probe) was added and further incubated for 2 hours before measuring absorbance at 570 nm and 600 nm using a 96-well plate reader. Cell proliferation was calculated following manufacturer provided formula. For apoptosis assay, 100,000 cells were seeded in a 12-well plate and incubated for 48 hours using three different conditions, with IL3, with FL, and without IL3 or FL. Apoptosis was measured by flow cytometry using Annexin V and 7-aminoactinomycin D (7-AAD) apoptosis kit (BD Biosciences). For colony formation assay, 1000 cells were mixed with 500 μl 80% methylcellulose medium and seeded in a 24-well plate. Cells were incubated for 7 days before counting colonies by two individual researchers.

### Microarray and patient data analysis

Ba/F3 and 32D cells expressing FLT3-ITD and empty vector or SLAP2 were washed three times and serum starved overnight before extracting total RNA using RNeasy mini kit (Qiagen). The quality of total RNA was checked by Bioanalyzer. Affymetrix GeneChip 2.0 ST array used for mRNA expression analysis. Raw data were processed for RMA normalization followed by oncogenic pathway enrichment analysis using Gene Set Enrichment Analysis (GSEA) software. Patient sample data were collected from previously published microarray dataset GSE14468 [32].

### Receptor degradation assay

Ba/F3 and 32D cells transfected with FLT3-WT and empty vector or SLAP2 were washed three times with PBS and treated with 100 μg/ml cycloheximide for 30 minutes followed by FL stimulation for 30 minutes. Cells were then washed and lysed using Triton X-100 lysis buffer. Cell lysates were used for Western blotting.

### Animal experiments

Four-week-old male BALB/c nude mice were used for xenograft experiments. Five mice in each group were injected subcutaneously with 0.1 ml PBS and Matrigel (1:1) containing 2 × 10^6^ control or SLAP2 expressing Ba/F3-FLT3-ITD and 32D-FLT3-ITD cells. Animals were monitored for weight change and tumor size. All mice were maintained following Hong Kong animal ethical regulation.

### Statistical analysis

Statistical analysis was done using GraphPad Prism 5.0. Data were expressed as the mean ± SE. Students *t-test* and one-way ANOVA with Bonferroni's pos *t-tests* were used.
